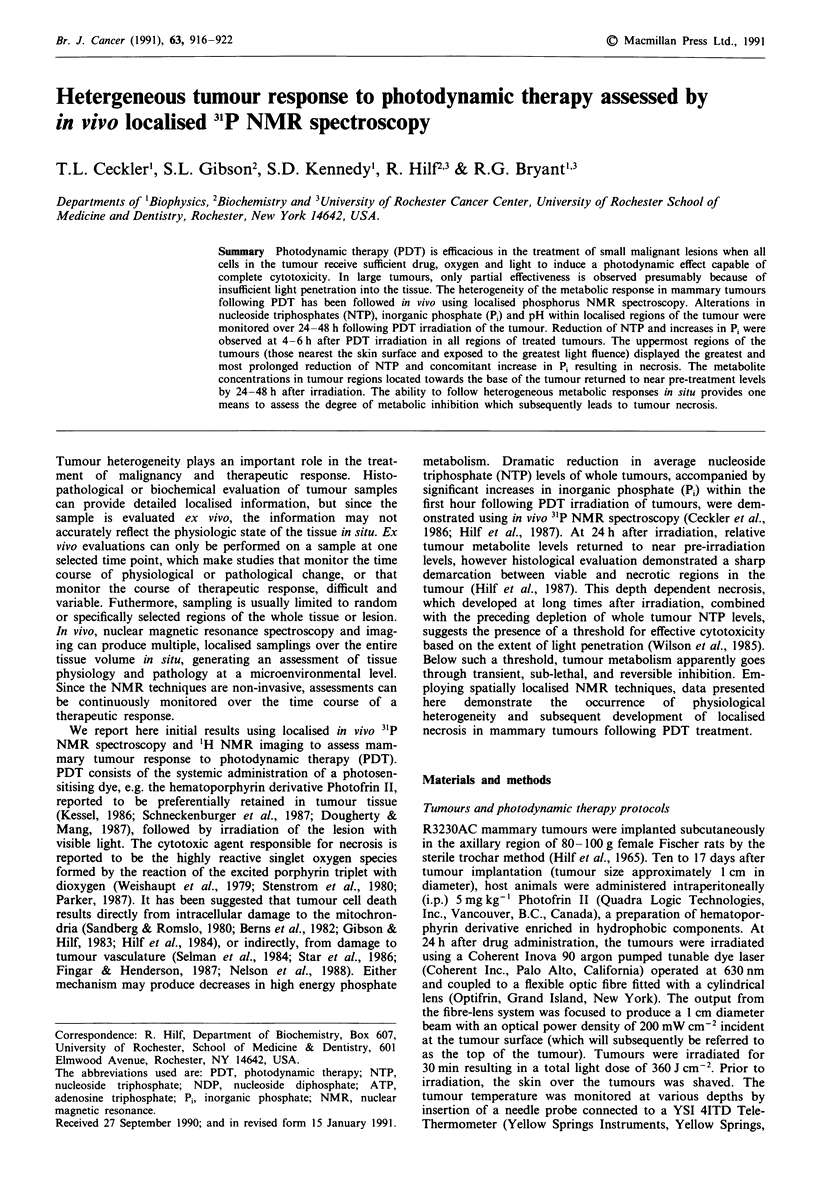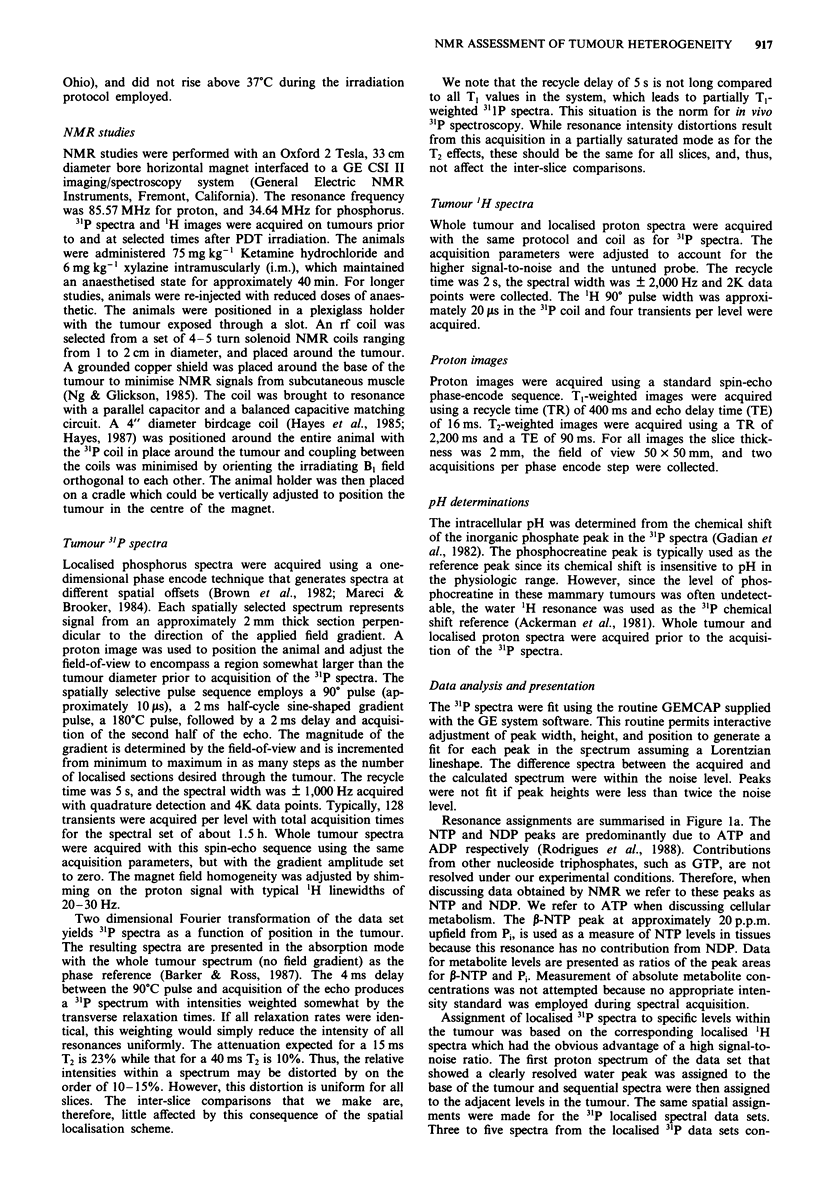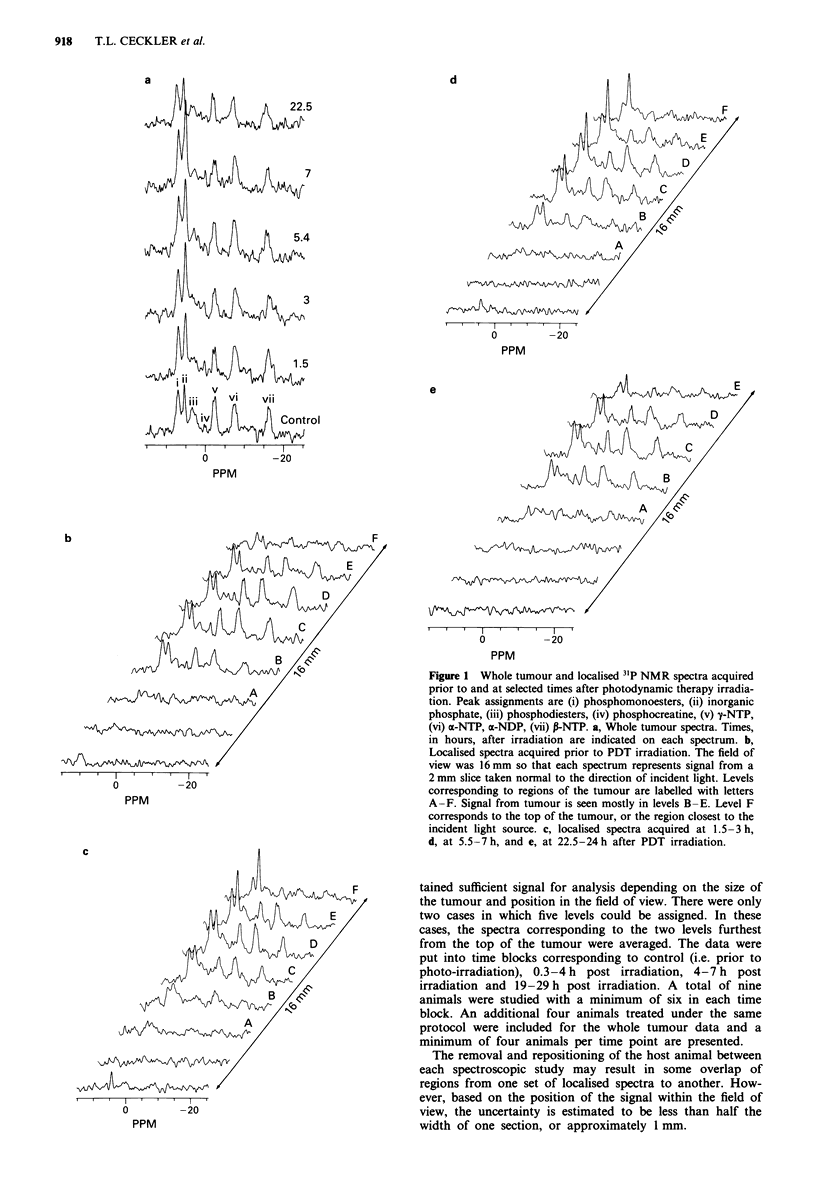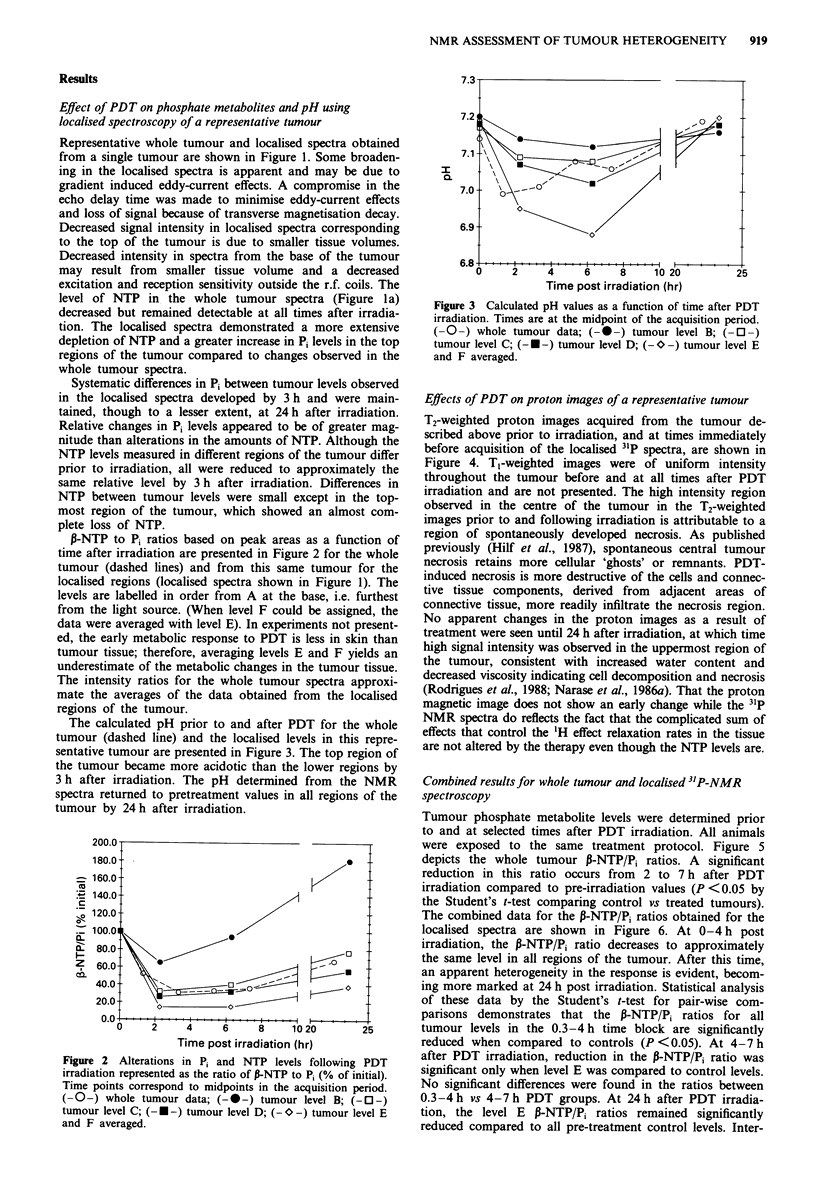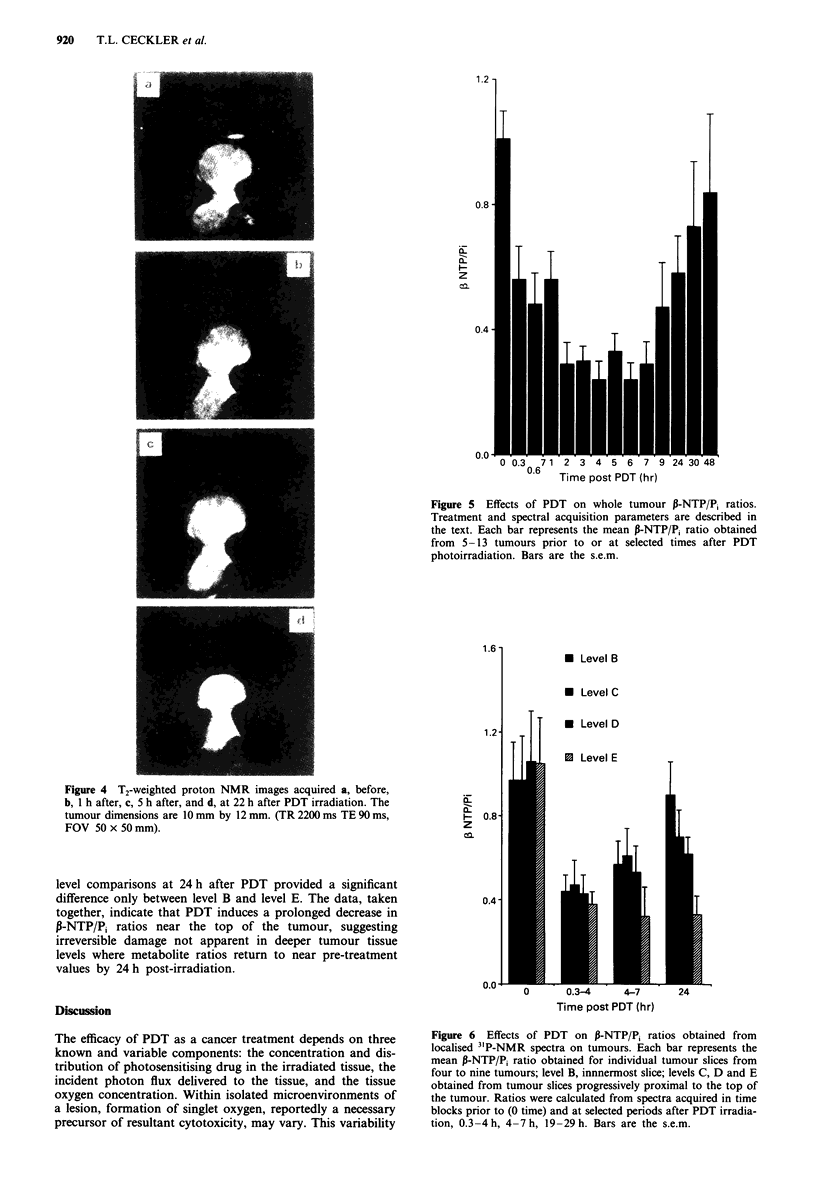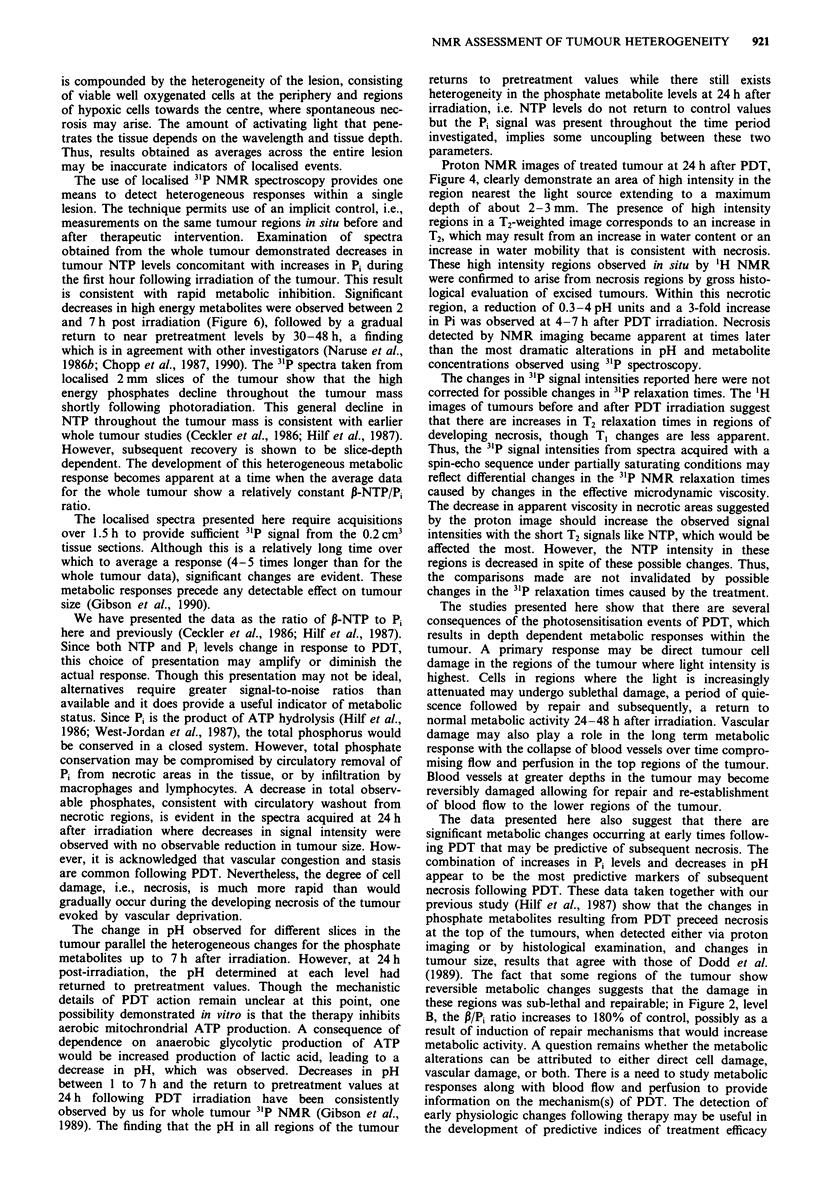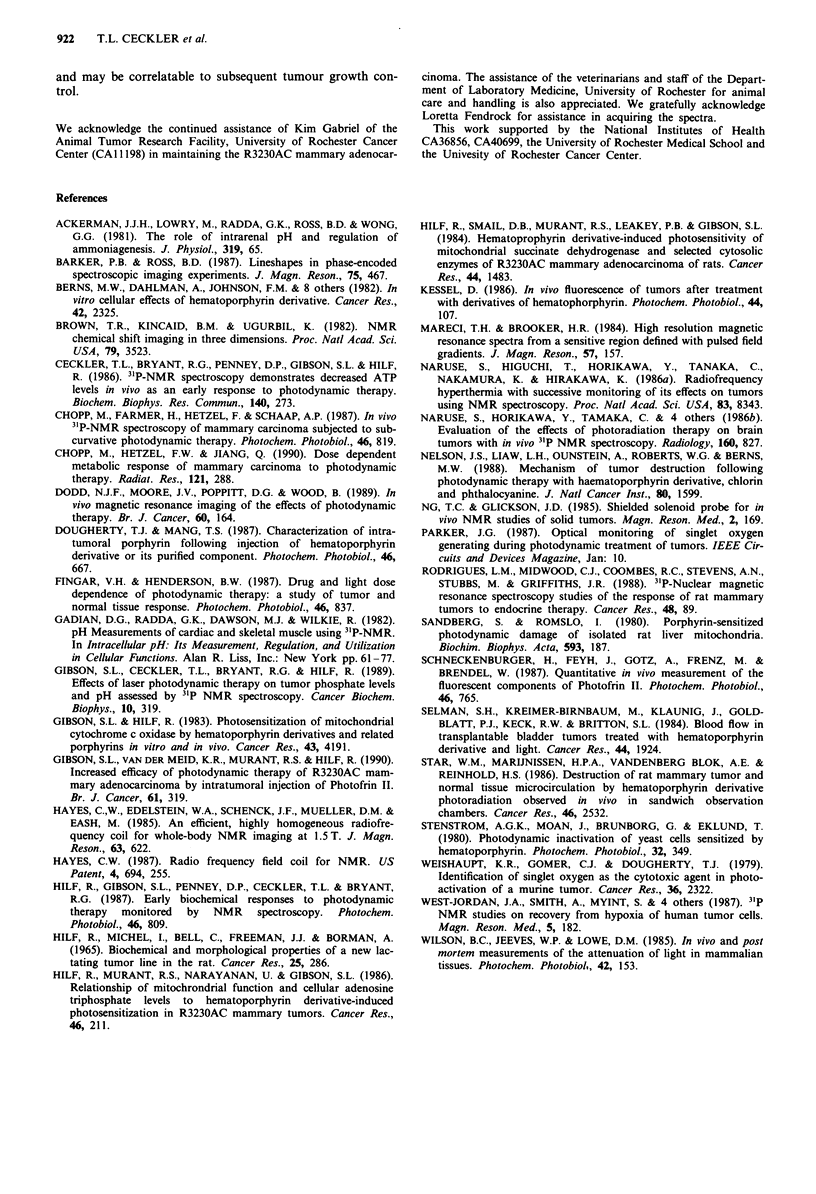# Hetergeneous tumour response to photodynamic therapy assessed by in vivo localised 31P NMR spectroscopy.

**DOI:** 10.1038/bjc.1991.201

**Published:** 1991-06

**Authors:** T. L. Ceckler, S. L. Gibson, S. D. Kennedy, R. Hill, R. G. Bryant

**Affiliations:** Department of Biophysics, University of Rochester School of Medicine and Dentistry, New York 14642.

## Abstract

**Images:**


Br. J. Cancer (1991), 63, 916–922 © Macmillan Press Ltd., 1991

### Hetergeneous tumour response to photodynamic therapy assessed by *in vivo* localised $^{31}P$ NMR spectroscopy

T.L. Ceckler[1], S.L. Gibson[2], S.D. Kennedy[1], R. Hilf[2,3] & R.G. Bryant[1,3]

*Departments of [1]Biophysics, [2]Biochemistry and [3]University of Rochester Cancer Center, University of Rochester School of Medicine and Dentistry, Rochester, New York 14642, USA.*

**Summary** Photodynamic therapy (PDT) is efficacious in the treatment of small malignant lesions when all cells in the tumour receive sufficient drug, oxygen and light to induce a photodynamic effect capable of complete cytotoxicity. In large tumours, only partial effectiveness is observed presumably because of insufficient light penetration into the tissue. The heterogeneity of the metabolic response in mammary tumours following PDT has been followed *in vivo* using localised phosphorus NMR spectroscopy. Alterations in nucleoside triphosphates (NTP), inorganic phosphate ($P_i$) and pH within localised regions of the tumour were monitored over 24–48 h following PDT irradiation of the tumour. Reduction of NTP and increases in $P_i$ were observed at 4–6 h after PDT irradiation in all regions of treated tumours. The uppermost regions of the tumours (those nearest the skin surface and exposed to the greatest light fluence) displayed the greatest and most prolonged reduction of NTP and concomitant increase in $P_i$ resulting in necrosis. The metabolite concentrations in tumour regions located towards the base of the tumour returned to near pre-treatment levels by 24–48 h after irradiation. The ability to follow heterogeneous metabolic responses *in situ* provides one means to assess the degree of metabolic inhibition which subsequently leads to tumour necrosis.

Tumour heterogeneity plays an important role in the treatment of malignancy and therapeutic response. Histopathological or biochemical evaluation of tumour samples can provide detailed localised information, but since the sample is evaluated *ex vivo*, the information may not accurately reflect the physiologic state of the tissue *in situ*. *Ex vivo* evaluations can only be performed on a sample at one selected time point, which make studies that monitor the time course of physiological or pathological change, or that monitor the course of therapeutic response, difficult and variable. Futhermore, sampling is usually limited to random or specifically selected regions of the whole tissue or lesion. *In vivo*, nuclear magnetic resonance spectroscopy and imaging can produce multiple, localised samplings over the entire tissue volume *in situ*, generating an assessment of tissue physiology and pathology at a microenvironmental level. Since the NMR techniques are non-invasive, assessments can be continuously monitored over the time course of a therapeutic response.

We report here initial results using localised *in vivo* $^{31}P$ NMR spectroscopy and $^{1}H$ NMR imaging to assess mammary tumour response to photodynamic therapy (PDT). PDT consists of the systemic administration of a photosensitising dye, e.g. the hematoporphyrin derivative Photofrin II, reported to be preferentially retained in tumour tissue (Kessel, 1986; Schneckenburger *et al.*, 1987; Dougherty & Mang, 1987), followed by irradiation of the lesion with visible light. The cytotoxic agent responsible for necrosis is reported to be the highly reactive singlet oxygen species formed by the reaction of the excited porphyrin triplet with dioxygen (Weishaupt *et al.*, 1979; Stenstrom *et al.*, 1980; Parker, 1987). It has been suggested that tumour cell death results directly from intracellular damage to the mitochrondria (Sandberg & Romslo, 1980; Berns *et al.*, 1982; Gibson & Hilf, 1983; Hilf *et al.*, 1984), or indirectly, from damage to tumour vasculature (Selman *et al.*, 1984; Star *et al.*, 1986; Fingar & Henderson, 1987; Nelson *et al.*, 1988). Either mechanism may produce decreases in high energy phosphate metabolism. Dramatic reduction in average nucleoside triphosphate (NTP) levels of whole tumours, accompanied by significant increases in inorganic phosphate ($P_i$) within the first hour following PDT irradiation of tumours, were demonstrated using *in vivo* $^{31}P$ NMR spectroscopy (Ceckler *et al.*, 1986; Hilf *et al.*, 1987). At 24 h after irradiation, relative tumour metabolite levels returned to near pre-irradiation levels, however histological evaluation demonstrated a sharp demarcation between viable and necrotic regions in the tumour (Hilf *et al.*, 1987). This depth dependent necrosis, which developed at long times after irradiation, combined with the preceding depletion of whole tumour NTP levels, suggests the presence of a threshold for effective cytotoxicity based on the extent of light penetration (Wilson *et al.*, 1985). Below such a threshold, tumour metabolism apparently goes through transient, sub-lethal, and reversible inhibition. Employing spatially localised NMR techniques, data presented here demonstrate the occurrence of physiological heterogeneity and subsequent development of localised necrosis in mammary tumours following PDT treatment.

### Materials and methods

### *Tumours and photodynamic therapy protocols*

R3230AC mammary tumours were implanted subcutaneously in the axillary region of 80–100 g female Fischer rats by the sterile trochar method (Hilf *et al.*, 1965). Ten to 17 days after tumour implantation (tumour size approximately 1 cm in diameter), host animals were administered intraperitoneally (i.p.) 5 mg $kg^{-1}$ Photofrin II (Quadra Logic Technologies, Inc., Vancouver, B.C., Canada), a preparation of hematoporphyrin derivative enriched in hydrophobic components. At 24 h after drug administration, the tumours were irradiated using a Coherent Inova 90 argon pumped tunable dye laser (Coherent Inc., Palo Alto, California) operated at 630 nm and coupled to a flexible optic fibre fitted with a cylindrical lens (Optifrin, Grand Island, New York). The output from the fibre-lens system was focused to produce a 1 cm diameter beam with an optical power density of 200 mW $cm^{-2}$ incident at the tumour surface (which will subsequently be referred to as the top of the tumour). Tumours were irradiated for 30 min resulting in a total light dose of 360 J $cm^{-2}$. Prior to irradiation, the skin over the tumours was shaved. The tumour temperature was monitored at various depths by insertion of a needle probe connected to a YSI 4ITD Tele-Thermometer (Yellow Springs Instruments, Yellow Springs,

Correspondence: R. Hilf, Department of Biochemistry, Box 607, University of Rochester, School of Medicine & Dentistry, 601 Elmwood Avenue, Rochester, NY 14642, USA.

The abbreviations used are: PDT, photodynamic therapy; NTP, nucleoside triphosphate; NDP, nucleoside diphosphate; ATP, adenosine triphosphate; $P_i$, inorganic phosphate; NMR, nuclear magnetic resonance.

Received 27 September 1990; and in revised form 15 January 1991.

Ohio), and did not rise above 37°C during the irradiation protocol employed.

*NMR studies*

NMR studies were performed with an Oxford 2 Tesla, 33 cm diameter bore horizontal magnet interfaced to a GE CSI II imaging/spectroscopy system (General Electric NMR Instruments, Fremont, California). The resonance frequency was 85.57 MHz for proton, and 34.64 MHz for phosphorus.

$^{31}$P spectra and $^{1}$H images were acquired on tumours prior to and at selected times after PDT irradiation. The animals were administered 75 mg kg$^{-1}$ Ketamine hydrochloride and 6 mg kg$^{-1}$ xylazine intramuscularly (i.m.), which maintained an anaesthetised state for approximately 40 min. For longer studies, animals were re-injected with reduced doses of anaesthetic. The animals were positioned in a plexiglass holder with the tumour exposed through a slot. An rf coil was selected from a set of 4–5 turn solenoid NMR coils ranging from 1 to 2 cm in diameter, and placed around the tumour. A grounded copper shield was placed around the base of the tumour to minimise NMR signals from subcutaneous muscle (Ng & Glickson, 1985). The coil was brought to resonance with a parallel capacitor and a balanced capacitive matching circuit. A 4" diameter birdcage coil (Hayes *et al*., 1985; Hayes, 1987) was positioned around the entire animal with the $^{31}$P coil in place around the tumour and coupling between the coils was minimised by orienting the irradiating $B_1$ field orthogonal to each other. The animal holder was then placed on a cradle which could be vertically adjusted to position the tumour in the centre of the magnet.

*Tumour $^{31}$P spectra*

Localised phosphorus spectra were acquired using a one-dimensional phase encode technique that generates spectra at different spatial offsets (Brown *et al*., 1982; Mareci & Brooker, 1984). Each spatially selected spectrum represents signal from an approximately 2 mm thick section perpendicular to the direction of the applied field gradient. A proton image was used to position the animal and adjust the field-of-view to encompass a region somewhat larger than the tumour diameter prior to acquisition of the $^{31}$P spectra. The spatially selective pulse sequence employs a 90° pulse (approximately 10 μs), a 2 ms half-cycle sine-shaped gradient pulse, a 180°C pulse, followed by a 2 ms delay and acquisition of the second half of the echo. The magnitude of the gradient is determined by the field-of-view and is incremented from minimum to maximum in as many steps as the number of localised sections desired through the tumour. The recycle time was 5 s, and the spectral width was ± 1,000 Hz acquired with quadrature detection and 4K data points. Typically, 128 transients were acquired per level with total acquisition times for the spectral set of about 1.5 h. Whole tumour spectra were acquired with this spin-echo sequence using the same acquisition parameters, but with the gradient amplitude set to zero. The magnet field homogeneity was adjusted by shimming on the proton signal with typical $^{1}$H linewidths of 20–30 Hz.

Two dimensional Fourier transformation of the data set yields $^{31}$P spectra as a function of position in the tumour. The resulting spectra are presented in the absorption mode with the whole tumour spectrum (no field gradient) as the phase reference (Barker & Ross, 1987). The 4 ms delay between the 90°C pulse and acquisition of the echo produces a $^{31}$P spectrum with intensities weighted somewhat by the transverse relaxation times. If all relaxation rates were identical, this weighting would simply reduce the intensity of all resonances uniformly. The attenuation expected for a 15 ms $T_2$ is 23% while that for a 40 ms $T_2$ is 10%. Thus, the relative intensities within a spectrum may be distorted by on the order of 10–15%. However, this distortion is uniform for all slices. The inter-slice comparisons that we make are, therefore, little affected by this consequence of the spatial localisation scheme.

We note that the recycle delay of 5 s is not long compared to all $T_1$ values in the system, which leads to partially $T_1$-weighted $^{31}$1P spectra. This situation is the norm for *in vivo* $^{31}$P spectroscopy. While resonance intensity distortions result from this acquisition in a partially saturated mode as for the $T_2$ effects, these should be the same for all slices, and, thus, not affect the inter-slice comparisons.

*Tumour $^{1}$H spectra*

Whole tumour and localised proton spectra were acquired with the same protocol and coil as for $^{31}$P spectra. The acquisition parameters were adjusted to account for the higher signal-to-noise and the untuned probe. The recycle time was 2 s, the spectral width was ± 2,000 Hz and 2K data points were collected. The $^{1}$H 90° pulse width was approximately 20 μs in the $^{31}$P coil and four transients per level were acquired.

*Proton images*

Proton images were acquired using a standard spin-echo phase-encode sequence. $T_1$-weighted images were acquired using a recycle time (TR) of 400 ms and echo delay time (TE) of 16 ms. $T_2$-weighted images were acquired using a TR of 2,200 ms and a TE of 90 ms. For all images the slice thickness was 2 mm, the field of view 50 × 50 mm, and two acquisitions per phase encode step were collected.

*pH determinations*

The intracellular pH was determined from the chemical shift of the inorganic phosphate peak in the $^{31}$P spectra (Gadian *et al*., 1982). The phosphocreatine peak is typically used as the reference peak since its chemical shift is insensitive to pH in the physiologic range. However, since the level of phosphocreatine in these mammary tumours was often undetectable, the water $^{1}$H resonance was used as the $^{31}$P chemical shift reference (Ackerman *et al*., 1981). Whole tumour and localised proton spectra were acquired prior to the acquisition of the $^{31}$P spectra.

*Data analysis and presentation*

The $^{31}$P spectra were fit using the routine GEMCAP supplied with the GE system software. This routine permits interactive adjustment of peak width, height, and position to generate a fit for each peak in the spectrum assuming a Lorentzian lineshape. The difference spectra between the acquired and the calculated spectrum were within the noise level. Peaks were not fit if peak heights were less than twice the noise level.

Resonance assignments are summarised in Figure 1a. The NTP and NDP peaks are predominantly due to ATP and ADP respectively (Rodrigues *et al*., 1988). Contributions from other nucleoside triphosphates, such as GTP, are not resolved under our experimental conditions. Therefore, when discussing data obtained by NMR we refer to these peaks as NTP and NDP. We refer to ATP when discussing cellular metabolism. The β-NTP peak at approximately 20 p.p.m. upfield from $P_i$, is used as a measure of NTP levels in tissues because this resonance has no contribution from NDP. Data for metabolite levels are presented as ratios of the peak areas for β-NTP and $P_i$. Measurement of absolute metabolite concentrations was not attempted because no appropriate intensity standard was employed during spectral acquisition.

Assignment of localised $^{31}$P spectra to specific levels within the tumour was based on the corresponding localised $^{1}$H spectra which had the obvious advantage of a high signal-to-noise ratio. The first proton spectrum of the data set that showed a clearly resolved water peak was assigned to the base of the tumour and sequential spectra were then assigned to the adjacent levels in the tumour. The same spatial assignments were made for the $^{31}$P localised spectral data sets. Three to five spectra from the localised $^{31}$P data sets con-

**Figure 1** Whole tumour and localised $^{31}P$ NMR spectra acquired prior to and at selected times after photodynamic therapy irradiation. Peak assignments are (i) phosphomonoesters, (ii) inorganic phosphate, (iii) phosphodiesters, (iv) phosphocreatine, (v) γ-NTP, (vi) α-NTP, α-NDP, (vii) β-NTP. **a**, Whole tumour spectra. Times, in hours, after irradiation are indicated on each spectrum. **b**, Localised spectra acquired prior to PDT irradiation. The field of view was 16 mm so that each spectrum represents signal from a 2 mm slice taken normal to the direction of incident light. Levels corresponding to regions of the tumour are labelled with letters A–F. Signal from tumour is seen mostly in levels B–E. Level F corresponds to the top of the tumour, or the region closest to the incident light source. **c**, localised spectra acquired at 1.5–3 h, **d**, at 5.5–7 h, and **e**, at 22.5–24 h after PDT irradiation.

tained sufficient signal for analysis depending on the size of the tumour and position in the field of view. There were only two cases in which five levels could be assigned. In these cases, the spectra corresponding to the two levels furthest from the top of the tumour were averaged. The data were put into time blocks corresponding to control (i.e. prior to photo-irradiation), 0.3–4 h post irradiation, 4–7 h post irradiation and 19–29 h post irradiation. A total of nine animals were studied with a minimum of six in each time block. An additional four animals treated under the same protocol were included for the whole tumour data and a minimum of four animals per time point are presented.

The removal and repositioning of the host animal between each spectroscopic study may result in some overlap of regions from one set of localised spectra to another. However, based on the position of the signal within the field of view, the uncertainty is estimated to be less than half the width of one section, or approximately 1 mm.

### Results

*Effect of PDT on phosphate metabolites and pH using localised spectroscopy of a representative tumour*

Representative whole tumour and localised spectra obtained from a single tumour are shown in Figure 1. Some broadening in the localised spectra is apparent and may be due to gradient induced eddy-current effects. A compromise in the echo delay time was made to minimise eddy-current effects and loss of signal because of transverse magnetisation decay. Decreased signal intensity in localised spectra corresponding to the top of the tumour is due to smaller tissue volumes. Decreased intensity in spectra from the base of the tumour may result from smaller tissue volume and a decreased excitation and reception sensitivity outside the r.f. coils. The level of NTP in the whole tumour spectra (Figure 1a) decreased but remained detectable at all times after irradiation. The localised spectra demonstrated a more extensive depletion of NTP and a greater increase in $P_i$ levels in the top regions of the tumour compared to changes observed in the whole tumour spectra.

Systematic differences in $P_i$ between tumour levels observed in the localised spectra developed by 3 h and were maintained, though to a lesser extent, at 24 h after irradiation. Relative changes in $P_i$ levels appeared to be of greater magnitude than alterations in the amounts of NTP. Although the NTP levels measured in different regions of the tumour differ prior to irradiation, all were reduced to approximately the same relative level by 3 h after irradiation. Differences in NTP between tumour levels were small except in the topmost region of the tumour, which showed an almost complete loss of NTP.

$\beta$-NTP to $P_i$ ratios based on peak areas as a function of time after irradiation are presented in Figure 2 for the whole tumour (dashed lines) and from this same tumour for the localised regions (localised spectra shown in Figure 1). The levels are labelled in order from A at the base, i.e. furthest from the light source. (When level F could be assigned, the data were averaged with level E). In experiments not presented, the early metabolic response to PDT is less in skin than tumour tissue; therefore, averaging levels E and F yields an underestimate of the metabolic changes in the tumour tissue. The intensity ratios for the whole tumour spectra approximate the averages of the data obtained from the localised regions of the tumour.

The calculated pH prior to and after PDT for the whole tumour (dashed line) and the localised levels in this representative tumour are presented in Figure 3. The top region of the tumour became more acidotic than the lower regions by 3 h after irradiation. The pH determined from the NMR spectra returned to pretreatment values in all regions of the tumour by 24 h after irradiation.

**Figure 2** Alterations in $P_i$ and NTP levels following PDT irradiation represented as the ratio of $\beta$-NTP to $P_i$ (% of initial). Time points correspond to midpoints in the acquisition period. (–○–) whole tumour data; (–●–) tumour level B; (–□–) tumour level C; (–■–) tumour level D; (–◇–) tumour level E and F averaged.

**Figure 3** Calculated pH values as a function of time after PDT irradiation. Times are at the midpoint of the acquisition period. (–○–) whole tumour data; (–●–) tumour level B; (–□–) tumour level C; (–■–) tumour level D; (–◇–) tumour level E and F averaged.

*Effects of PDT on proton images of a representative tumour*

$T_2$-weighted proton images acquired from the tumour described above prior to irradiation, and at times immediately before acquisition of the localised $^{31}P$ spectra, are shown in Figure 4. $T_1$-weighted images were of uniform intensity throughout the tumour before and at all times after PDT irradiation and are not presented. The high intensity region observed in the centre of the tumour in the $T_2$-weighted images prior to and following irradiation is attributable to a region of spontaneously developed necrosis. As published previously (Hilf *et al.*, 1987), spontaneous central tumour necrosis retains more cellular 'ghosts' or remnants. PDT-induced necrosis is more destructive of the cells and connective tissue components, derived from adjacent areas of connective tissue, more readily infiltrate the necrosis region. No apparent changes in the proton images as a result of treatment were seen until 24 h after irradiation, at which time high signal intensity was observed in the uppermost region of the tumour, consistent with increased water content and decreased viscosity indicating cell decomposition and necrosis (Rodrigues *et al.*, 1988; Narase *et al.*, 1986*a*). That the proton magnetic image does not show an early change while the $^{31}P$ NMR spectra do reflects the fact that the complicated sum of effects that control the $^1H$ effect relaxation rates in the tissue are not altered by the therapy even though the NTP levels are.

*Combined results for whole tumour and localised $^{31}P$-NMR spectroscopy*

Tumour phosphate metabolite levels were determined prior to and at selected times after PDT irradiation. All animals were exposed to the same treatment protocol. Figure 5 depicts the whole tumour $\beta$-NTP/$P_i$ ratios. A significant reduction in this ratio occurs from 2 to 7 h after PDT irradiation compared to pre-irradiation values ($P < 0.05$ by the Student's $t$-test comparing control *vs* treated tumours). The combined data for the $\beta$-NTP/$P_i$ ratios obtained for the localised spectra are shown in Figure 6. At 0–4 h post irradiation, the $\beta$-NTP/$P_i$ ratio decreases to approximately the same level in all regions of the tumour. After this time, an apparent heterogeneity in the response is evident, becoming more marked at 24 h post irradiation. Statistical analysis of these data by the Student's $t$-test for pair-wise comparisons demonstrates that the $\beta$-NTP/$P_i$ ratios for all tumour levels in the 0.3–4 h time block are significantly reduced when compared to controls ($P < 0.05$). At 4–7 h after PDT irradiation, reduction in the $\beta$-NTP/$P_i$ ratio was significant only when level E was compared to control levels. No significant differences were found in the ratios between 0.3–4 h *vs* 4–7 h PDT groups. At 24 h after PDT irradiation, the level E $\beta$-NTP/$P_i$ ratios remained significantly reduced compared to all pre-treatment control levels. Inter-

**Figure 4** $T_2$-weighted proton NMR images acquired **a**, before, **b**, 1 h after, **c**, 5 h after, and **d**, at 22 h after PDT irradiation. The tumour dimensions are 10 mm by 12 mm. (TR 2200 ms TE 90 ms, FOV 50 × 50 mm).

**Figure 5** Effects of PDT on whole tumour $\beta$-NTP/$P_i$ ratios. Treatment and spectral acquisition parameters are described in the text. Each bar represents the mean $\beta$-NTP/$P_i$ ratio obtained from 5–13 tumours prior to or at selected times after PDT photoirradiation. Bars are the s.e.m.

**Figure 6** Effects of PDT on $\beta$-NTP/$P_i$ ratios obtained from localised $^{31}$P-NMR spectra on tumours. Each bar represents the mean $\beta$-NTP/$P_i$ ratio obtained for individual tumour slices from four to nine tumours; level B, innnermost slice; levels C, D and E obtained from tumour slices progressively proximal to the top of the tumour. Ratios were calculated from spectra acquired in time blocks prior to (0 time) and at selected periods after PDT irradiation, 0.3–4 h, 4–7 h, 19–29 h. Bars are the s.e.m.

level comparisons at 24 h after PDT provided a significant difference only between level B and level E. The data, taken together, indicate that PDT induces a prolonged decrease in $\beta$-NTP/$P_i$ ratios near the top of the tumour, suggesting irreversible damage not apparent in deeper tumour tissue levels where metabolite ratios return to near pre-treatment values by 24 h post-irradiation.

### Discussion

The efficacy of PDT as a cancer treatment depends on three known and variable components: the concentration and distribution of photosensitising drug in the irradiated tissue, the incident photon flux delivered to the tissue, and the tissue oxygen concentration. Within isolated microenvironments of a lesion, formation of singlet oxygen, reportedly a necessary precursor of resultant cytotoxicity, may vary. This variability is compounded by the heterogeneity of the lesion, consisting of viable well oxygenated cells at the periphery and regions of hypoxic cells towards the centre, where spontaneous necrosis may arise. The amount of activating light that penetrates the tissue depends on the wavelength and tissue depth. Thus, results obtained as averages across the entire lesion may be inaccurate indicators of localised events.

The use of localised $^{31}$P NMR spectroscopy provides one means to detect heterogeneous responses within a single lesion. The technique permits use of an implicit control, i.e., measurements on the same tumour regions *in situ* before and after therapeutic intervention. Examination of spectra obtained from the whole tumour demonstrated decreases in tumour NTP levels concomitant with increases in $P_i$ during the first hour following irradiation of the tumour. This result is consistent with rapid metabolic inhibition. Significant decreases in high energy metabolites were observed between 2 and 7 h post irradiation (Figure 6), followed by a gradual return to near pretreatment levels by 30–48 h, a finding which is in agreement with other investigators (Naruse *et al.*, 1986*b*; Chopp *et al.*, 1987, 1990). The $^{31}$P spectra taken from localised 2 mm slices of the tumour show that the high energy phosphates decline throughout the tumour mass shortly following photoradiation. This general decline in NTP throughout the tumour mass is consistent with earlier whole tumour studies (Ceckler *et al.*, 1986; Hilf *et al.*, 1987). However, subsequent recovery is shown to be slice-depth dependent. The development of this heterogeneous metabolic response becomes apparent at a time when the average data for the whole tumour show a relatively constant $\beta$-NTP/$P_i$ ratio.

The localised spectra presented here require acquisitions over 1.5 h to provide sufficient $^{31}$P signal from the 0.2 cm$^3$ tissue sections. Although this is a relatively long time over which to average a response (4–5 times longer than for the whole tumour data), significant changes are evident. These metabolic responses precede any detectable effect on tumour size (Gibson *et al.*, 1990).

We have presented the data as the ratio of $\beta$-NTP to $P_i$ here and previously (Ceckler *et al.*, 1986; Hilf *et al.*, 1987). Since both NTP and $P_i$ levels change in response to PDT, this choice of presentation may amplify or diminish the actual response. Though this presentation may not be ideal, alternatives require greater signal-to-noise ratios than available and it does provide a useful indicator of metabolic status. Since $P_i$ is the product of ATP hydrolysis (Hilf *et al.*, 1986; West-Jordan *et al.*, 1987), the total phosphorus would be conserved in a closed system. However, total phosphate conservation may be compromised by circulatory removal of $P_i$ from necrotic areas in the tissue, or by infiltration by macrophages and lymphocytes. A decrease in total observable phosphates, consistent with circulatory washout from necrotic regions, is evident in the spectra acquired at 24 h after irradiation where decreases in signal intensity were observed with no observable reduction in tumour size. However, it is acknowledged that vascular congestion and stasis are common following PDT. Nevertheless, the degree of cell damage, i.e., necrosis, is much more rapid than would gradually occur during the developing necrosis of the tumour evoked by vascular deprivation.

The change in pH observed for different slices in the tumour parallel the heterogeneous changes for the phosphate metabolites up to 7 h after irradiation. However, at 24 h post-irradiation, the pH determined at each level had returned to pretreatment values. Though the mechanistic details of PDT action remain unclear at this point, one possibility demonstrated *in vitro* is that the therapy inhibits aerobic mitochrondrial ATP production. A consequence of dependence on anaerobic glycolytic production of ATP would be increased production of lactic acid, leading to a decrease in pH, which was observed. Decreases in pH between 1 to 7 h and the return to pretreatment values at 24 h following PDT irradiation have been consistently observed by us for whole tumour $^{31}$P NMR (Gibson *et al.*, 1989). The finding that the pH in all regions of the tumour returns to pretreatment values while there still exists heterogeneity in the phosphate metabolite levels at 24 h after irradiation, i.e. NTP levels do not return to control values but the $P_i$ signal was present throughout the time period investigated, implies some uncoupling between these two parameters.

Proton NMR images of treated tumour at 24 h after PDT, Figure 4, clearly demonstrate an area of high intensity in the region nearest the light source extending to a maximum depth of about 2–3 mm. The presence of high intensity regions in a $T_2$-weighted image corresponds to an increase in $T_2$, which may result from an increase in water content or an increase in water mobility that is consistent with necrosis. These high intensity regions observed *in situ* by $^1$H NMR were confirmed to arise from necrosis regions by gross histological evaluation of excised tumours. Within this necrotic region, a reduction of 0.3–4 pH units and a 3-fold increase in Pi was observed at 4–7 h after PDT irradiation. Necrosis detected by NMR imaging became apparent at times later than the most dramatic alterations in pH and metabolite concentrations observed using $^{31}$P spectroscopy.

The changes in $^{31}$P signal intensities reported here were not corrected for possible changes in $^{31}$P relaxation times. The $^1$H images of tumours before and after PDT irradiation suggest that there are increases in $T_2$ relaxation times in regions of developing necrosis, though $T_1$ changes are less apparent. Thus, the $^{31}$P signal intensities from spectra acquired with a spin-echo sequence under partially saturating conditions may reflect differential changes in the $^{31}$P NMR relaxation times caused by changes in the effective microdynamic viscosity. The decrease in apparent viscosity in necrotic areas suggested by the proton image should increase the observed signal intensities with the short $T_2$ signals like NTP, which would be affected the most. However, the NTP intensity in these regions is decreased in spite of these possible changes. Thus, the comparisons made are not invalidated by possible changes in the $^{31}$P relaxation times caused by the treatment.

The studies presented here show that there are several consequences of the photosensitisation events of PDT, which results in depth dependent metabolic responses within the tumour. A primary response may be direct tumour cell damage in the regions of the tumour where light intensity is highest. Cells in regions where the light is increasingly attenuated may undergo sublethal damage, a period of quiescence followed by repair and subsequently, a return to normal metabolic activity 24–48 h after irradiation. Vascular damage may also play a role in the long term metabolic response with the collapse of blood vessels over time compromising flow and perfusion in the top regions of the tumour. Blood vessels at greater depths in the tumour may become reversibly damaged allowing for repair and re-establishment of blood flow to the lower regions of the tumour.

The data presented here also suggest that there are significant metabolic changes occurring at early times following PDT that may be predictive of subsequent necrosis. The combination of increases in $P_i$ levels and decreases in pH appear to be the most predictive markers of subsequent necrosis following PDT. These data taken together with our previous study (Hilf *et al.*, 1987) show that the changes in phosphate metabolites resulting from PDT preceed necrosis at the top of the tumours, when detected either via proton imaging or by histological examination, and changes in tumour size, results that agree with those of Dodd *et al.* (1989). The fact that some regions of the tumour show reversible metabolic changes suggests that the damage in these regions was sub-lethal and repairable; in Figure 2, level B, the $\beta$/$P_i$ ratio increases to 180% of control, possibly as a result of induction of repair mechanisms that would increase metabolic activity. A question remains whether the metabolic alterations can be attributed to either direct cell damage, vascular damage, or both. There is a need to study metabolic responses along with blood flow and perfusion to provide information on the mechanism(s) of PDT. The detection of early physiologic changes following therapy may be useful in the development of predictive indices of treatment efficacy and may be correlatable to subsequent tumour growth control.

We acknowledge the continued assistance of Kim Gabriel of the Animal Tumor Research Facility, University of Rochester Cancer Center (CA11198) in maintaining the R3230AC mammary adenocarcinoma. The assistance of the veterinarians and staff of the Department of Laboratory Medicine, University of Rochester for animal care and handling is also appreciated. We gratefully acknowledge Loretta Fendrock for assistance in acquiring the spectra.

This work supported by the National Institutes of Health CA36856, CA40699, the University of Rochester Medical School and the Univesity of Rochester Cancer Center.

### References

ACKERMAN, J.J.H., LOWRY, M., RADDA, G.K., ROSS, B.D. & WONG, G.G. (1981). The role of intrarenal pH and regulation of ammoniagenesis. *J. Physiol.*, **319**, 65.

BARKER, P.B. & ROSS, B.D. (1987). Lineshapes in phase-encoded spectroscopic imaging experiments. *J. Magn. Reson.*, **75**, 467.

BERNS, M.W., DAHLMAN, A., JOHNSON, F.M. & 8 others (1982). *In vitro* cellular effects of hematoporphyrin derivative. *Cancer Res.*, **42**, 2325.

BROWN, T.R., KINCAID, B.M. & UGURBIL, K. (1982). NMR chemical shift imaging in three dimensions. *Proc. Natl Acad. Sci. USA*, **79**, 3523.

CECKLER, T.L., BRYANT, R.G., PENNEY, D.P., GIBSON, S.L. & HILF, R. (1986). $^{31}$P-NMR spectroscopy demonstrates decreased ATP levels *in vivo* as an early response to photodynamic therapy. *Biochem. Biophys. Res. Commun.*, **140**, 273.

CHOPP, M., FARMER, H., HETZEL, F. & SCHAAP, A.P. (1987). *In vivo* $^{31}$P-NMR spectroscopy of mammary carcinoma subjected to subcurvative photodynamic therapy. *Photochem. Photobiol.*, **46**, 819.

CHOPP, M., HETZEL, F.W. & JIANG, Q. (1990). Dose dependent metabolic response of mammary carcinoma to photodynamic therapy. *Radiat. Res.*, **121**, 288.

DODD, N.J.F., MOORE, J.V., POPPITT, D.G. & WOOD, B. (1989). *In vivo* magnetic resonance imaging of the effects of photodynamic therapy. *Br. J. Cancer*, **60**, 164.

DOUGHERTY, T.J. & MANG, T.S. (1987). Characterization of intratumoral porphyrin following injection of hematoporphyrin derivative or its purified component. *Photochem. Photobiol.*, **46**, 667.

FINGAR, V.H. & HENDERSON, B.W. (1987). Drug and light dose dependence of photodynamic therapy: a study of tumor and normal tissue response. *Photochem. Photobiol.*, **46**, 837.

GADIAN, D.G., RADDA, G.K., DAWSON, M.J. & WILKIE, R. (1982). pH Measurements of cardiac and skeletal muscle using $^{31}$P-NMR. In *Intracellular pH: Its Measurement, Regulation, and Utilization in Cellular Functions*. Alan R. Liss, Inc.: New York pp. 61–77.

GIBSON, S.L., CECKLER, T.L., BRYANT, R.G. & HILF, R. (1989). Effects of laser photodynamic therapy on tumor phosphate levels and pH assessed by $^{31}$P NMR spectroscopy. *Cancer Biochem. Biophys.*, **10**, 319.

GIBSON, S.L. & HILF, R. (1983). Photosensitization of mitochondrial cytochrome c oxidase by hematoporphyrin derivatives and related porphyrins *in vitro and in vivo*. *Cancer Res.*, **43**, 4191.

GIBSON, S.L., VAN DER MEID, K.R., MURANT, R.S. & HILF, R. (1990). Increased efficacy of photodynamic therapy of R3230AC mammary adenocarcinoma by intratumoral injection of Photofrin II. *Br. J. Cancer*, **61**, 319.

HAYES, C.,W., EDELSTEIN, W.A., SCHENCK, J.F., MUELLER, D.M. & EASH, M. (1985). An efficient, highly homogeneous radiofrequency coil for whole-body NMR imaging at 1.5 T. *J. Magn. Reson.*, **63**, 622.

HAYES, C.W. (1987). Radio frequency field coil for NMR. *US Patent*, **4**, 694, 255.

HILF, R., GIBSON, S.L., PENNEY, D.P., CECKLER, T.L. & BRYANT, R.G. (1987). Early biochemical responses to photodynamic therapy monitored by NMR spectroscopy. *Photochem. Photobiol.*, **46**, 809.

HILF, R., MICHEL, I., BELL, C., FREEMAN, J.J. & BORMAN, A. (1965). Biochemical and morphological properties of a new lactating tumor line in the rat. *Cancer Res.*, **25**, 286.

HILF, R., MURANT, R.S., NARAYANAN, U. & GIBSON, S.L. (1986). Relationship of mitochrondrial function and cellular adenosine triphosphate levels to hematoporphyrin derivative-induced photosensitization in R3230AC mammary tumors. *Cancer Res.*, **46**, 211.

HILF, R., SMAIL, D.B., MURANT, R.S., LEAKEY, P.B. & GIBSON, S.L. (1984). Hematoprophyrin derivative-induced photosensitivity of mitochondrial succinate dehydrogenase and selected cytosolic enzymes of R3230AC mammary adenocarcinoma of rats. *Cancer Res.*, **44**, 1483.

KESSEL, D. (1986). *In vivo* fluorescence of tumors after treatment with derivatives of hematophorphyrin. *Photochem. Photobiol.*, **44**, 107.

MARECI, T.H. & BROOKER, H.R. (1984). High resolution magnetic resonance spectra from a sensitive region defined with pulsed field gradients. *J. Magn. Reson.*, **57**, 157.

NARUSE, S., HIGUCHI, T., HORIKAWA, Y., TANAKA, C., NAKAMURA, K. & HIRAKAWA, K. (1986*a*). Radiofrequency hyperthermia with successive monitoring of its effects on tumors using NMR spectroscopy. *Proc. Natl Acad. Sci. USA*, **83**, 8343.

NARUSE, S., HORIKAWA, Y., TAMAKA, C. & 4 others (1986*b*). Evaluation of the effects of photoradiation therapy on brain tumors with *in vivo* $^{31}$P NMR spectroscopy. *Radiology*, **160**, 827.

NELSON, J.S., LIAW, L.H., OUNSTEIN, A., ROBERTS, W.G. & BERNS, M.W. (1988). Mechanism of tumor destruction following photodynamic therapy with haematoporphyrin derivative, chlorin and phthalocyanine. *J. Natl Cancer Inst.*, **80**, 1599.

NG, T.C. & GLICKSON, J.D. (1985). Shielded solenoid probe for *in vivo* NMR studies of solid tumors. *Magn. Reson. Med.*, **2**, 169.

PARKER, J.G. (1987). Optical monitoring of singlet oxygen generating during photodynamic treatment of tumors. *IEEE Circuits and Devices Magazine*, Jan: 10.

RODRIGUES, L.M., MIDWOOD, C.J., COOMBES, R.C., STEVENS, A.N., STUBBS, M. & GRIFFITHS, J.R. (1988). $^{31}$P-Nuclear magnetic resonance spectroscopy studies of the response of rat mammary tumors to endocrine therapy. *Cancer Res.*, **48**, 89.

SANDBERG, S. & ROMSLO, I. (1980). Porphyrin-sensitized photodynamic damage of isolated rat liver mitochondria. *Biochim. Biophys. Acta*, **593**, 187.

SCHNECKENBURGER, H., FEYH, J., GOTZ, A., FRENZ, M. & BRENDEL, W. (1987). Quantitative *in vivo* measurement of the fluorescent components of Photofrin II. *Photochem. Photobiol.*, **46**, 765.

SELMAN, S.H., KREIMER-BIRNBAUM, M., KLAUNIG, J., GOLDBLATT, P.J., KECK, R.W. & BRITTON, S.L. (1984). Blood flow in transplantable bladder tumors treated with hematoporphyrin derivative and light. *Cancer Res.*, **44**, 1924.

STAR, W.M., MARIJNISSEN, H.P.A., VANDENBERG BLOK, A.E. & REINHOLD, H.S. (1986). Destruction of rat mammary tumor and normal tissue microcirculation by hematoporphyrin derivative photoradiation observed *in vivo* in sandwich observation chambers. *Cancer Res.*, **46**, 2532.

STENSTROM, A.G.K., MOAN, J., BRUNBORG, G. & EKLUND, T. (1980). Photodynamic inactivation of yeast cells sensitized by hematoporphyrin. *Photochem. Photobiol.*, **32**, 349.

WEISHAUPT, K.R., GOMER, C.J. & DOUGHERTY, T.J. (1979). Identification of singlet oxygen as the cytotoxic agent in photoactivation of a murine tumor. *Cancer Res.*, **36**, 2322.

WEST-JORDAN, J.A., SMITH, A., MYINT, S. & 4 others (1987). $^{31}$P NMR studies on recovery from hypoxia of human tumor cells. *Magn. Reson. Med.*, **5**, 182.

WILSON, B.C., JEEVES, W.P. & LOWE, D.M. (1985). *In vivo* and *post mortem* measurements of the attenuation of light in mammalian tissues. *Photochem. Photobiol*, **42**, 153.